# CODIFI2: Randomised controlled trial to compare clinical and cost‐effectiveness of swabs versus tissue sampling to inform management of infected diabetic foot ulcers

**DOI:** 10.1111/dme.70009

**Published:** 2025-03-06

**Authors:** E. Andrea Nelson, Sarah T. Brown, Colin C. Everett, Angela Oates, Michael Backhouse, Howard Collier, Joanna Dennett, Rachael Gilberts, Ben Lipsky, Michelle M. Lister, Jane E. Nixon, David Russell, Tim Sloan, Fran Game

**Affiliations:** ^1^ Glasgow Caledonian University Glasgow UK; ^2^ CTRU, Faculty of Medicine and Health University of Leeds Leeds UK; ^3^ Leeds Beckett University Leeds UK; ^4^ Warwick Clinical Trials Unit University of Warwick Warwick UK; ^5^ University of Washington Seattle Washington USA; ^6^ Nottingham University Hospitals NHS Trust Nottingham UK; ^7^ Health Sciences, Faculty of Medicine and Health University of Leeds Leeds UK; ^8^ Faculty of Medicine and Health University of Leeds Leeds UK; ^9^ University of Nottingham Nottingham UK; ^10^ University Hospitals of Derby and Burton NHS Foundation Trust Derby UK

**Keywords:** clinical trials, diabetic foot, diagnosis, infection

## Abstract

**BAims/Hypothesis:**

CODIFI2 compared wound swabbing and tissue sampling in people with infected diabetic foot ulcers (DFU) to determine the effects on clinical outcomes.

**Methods:**

Multicentre, Phase III, prospective, non‐blind, 2‐arm parallel group, randomised controlled trial comparing time to ulcer healing (primary outcome), proportions healed, antimicrobial regimen, ulcer area reduction, hospitalisation duration, and time to death for swab compared to tissue sampling. Allocation was via a central and independent randomisation system, with minimisation by DFU site, number, type, size, location, and duration.

Follow‐up was 52–104 weeks, with healing confirmed by a blinded assessor. Samplesize target was 730 participants for 90% power to detect a 12.5% difference in healing at 52 weeks.

**Results:**

Between May 2019 and May 2022, 149 participants were recruited (75 Swab, 74 Tissue) from 21 UK sites. The 52‐week cumulative incidence of confirmed healing as the first event was 45.3% (33.5%–56.4%) and 44.6% (33.0–55.6%) for swab vs. tissue. The hazard ratio (HR) for healing for tissue vs. swab was 1.01 (95% CI 0.65–1.55). The median (IQR) days in hospital was 17 (12–39) for swab and 16 (10–32) for tissue. Seventeen swab and 7 tissue participants died during follow‐up, and 18.7% and 24.3% of participants in the swab and tissue groups, respectively, had an amputation.

**Conclusions/Interpretation:**

This trial was underpowered to determine whether swab or tissue sampling impacted the rate of healing or time to healing. Clinical prescribing and patient outcomes differed slightly between groups; hence, the clinical benefit of tissue sampling is not established.

**What is already known about this subject?**

- Tissue samples identify pathogens in more patients and report more pathogens, versus swabbing in diabetic foot ulcers.

**What this study found?**

- Tissue sampling (vs swab), in mild/moderately infected DFU, preceded a higher rate of revision of antibiotics at review and more antibiotics prescriptions.
- There was no evidence of a difference in time to healing, ulcer area reduction at 4 weeks, or rates of complete healing at 52 weeks between sampling approaches: adverse events and amputations were slightly higher in the tissue group.

**How might this impact on clinical practice in the foreseeable future?**

- Clinicians may choose swabbing over tissue sampling for mild–moderately infected DFU with no impact on healing and reduction in costs if these findings are replicated in future studies.

## 1 INTRODUCTION

Diabetic foot ulcers (DFUs) are a common complication of diabetes.^1^, ^2^ Ulcers are vulnerable to infection, with around 40% of recent‐onset DFUs clinically infected at presentation.^3^ Infection can lead to substantial morbidity, including amputation; hence, guidelines advise that people with a DFU should be assessed for infection at every visit.^4^

The diagnosis of DFU infection is not dependent upon microbiological sampling, but requires a clinical assessment of the person, their limb, and wound. Fever, exudate, redness, inflammation, pus, odour, and bleeding indicate infection, and because the risks associated with DFU infection are substantial, empiric antibiotic therapy is initiated promptly. Antimicrobial regimens follow local guidelines, using knowledge of probable causative organisms, with wound samples taken for microbiology to confirm antimicrobial cover and facilitate subsequent modifications of the empiric antibiotics.^5^, ^6^, ^7^

Clinicians often obtain ulcer specimens with a cotton swab, collecting fluid from the deep ulcer bed.^8^ Swabs are readily available; sampling is quick, easy, non‐invasive, and inexpensive. Clinical guidelines, however, recommend tissue sampling to detect more pathogens and reduce sampling of colonising bacteria.^4^, ^9^, ^10^ Tissue samples identify bacteria from DFU more frequently than swabs and report more isolates per sample.^5^, ^11^, ^12^ This could be beneficial if pathogens, rather than colonising bacteria, are found, potentially leading to more tailoring of therapeutics. Better sampling could potentially lead to more rapid resolution of infection and even quicker healing. It could also reduce the use of broad‐spectrum antibiotics, reducing antibiotic resistance.^13^, ^14^ Conversely, tissue sampling's higher yield might increase prescribing. It is unknown whether using tissue sampling or swab sampling leads to changes in prescribing in clinical settings or whether any such change in prescribing affects clinical outcomes. The aim of the CODIFI2 study was to compare wound swabbing and tissue sampling in people with infected diabetic foot ulcers (DFU) to determine effects on clinical outcomes.

## 2 METHODS

CODIFI2 (ISRCTN74929588; registered 8th January 2019) was a multicentre, Phase III, prospective, 2‐arm parallel group, randomised controlled trial comparing the effects on time to ulcer healing of sampling techniques (swab vs. tissue sampling), with blinded outcome assessment, in DFUs clinically assessed as having a mild or moderate infection. This paper reports the clinical results. The primary objective was to determine the clinical effectiveness of tissue vs. swab sampling in terms of ulcer healing, with secondary objectives reporting antibiotic prescribing, reduction in ulcer area, proportion of ulcers healing fully, and incidence of adverse events.

The study protocol was approved by the West of Scotland Research Ethics Committee (18/WS/0235). Informed consent was obtained from participants on the day they attended the clinic with a DFU clinically assessed as infected. CODIFI2 has been carried out in accordance with the principles of the Declaration of Helsinki as revised in 2008.

### 2.1 Participants and Setting

People with diabetic foot ulcers, aged at least 18 years, in which the clinician suspected mild/moderate soft tissue infection, as per IDSA guidelines,^6^ who provided written consent for the study and photography of the foot. Reasons for exclusion included index ulcers present for >2 years, suspected osteomyelitis or severe infection, unwilling or not expected to comply with follow‐up or the sampling strategy. Participants were recruited from UK NHS secondary care and community clinics providing multi‐disciplinary team DFU services.

### 2.2 Randomisation and blinding

Eligible and consenting participants were randomised using an automated, secure, central 24‐h randomisation service, accessed via the web or telephone, implemented and maintained by the University of Leeds Clinical Trials Research Unit (CTRU). Researchers at sites required authorisation codes and a participant's unique NHS number to randomise: re‐enrolment was not permitted. Participants were assigned a sequential patient identifier and allocated to swab or tissue sampling with a minimisation algorithm incorporating a random element (80% chance of allocation reducing imbalances) to ensure groups were similar with respect to recruiting centres, number of DFU(s) present at baseline (1 vs. 2 or more), DFU duration (<6 months vs. ≥6 months), ulcer area (<1 cm^2^ vs. ≥1 cm^2^), ulcer location (forefoot vs. midfoot/hindfoot) and aetiology (neuropathic, neuro‐ischaemic, or ischaemic). Allocation was disclosed to the user on randomisation, confirmed by email. Neither treating clinicians nor participants were blind to randomised allocations, as we deemed this not achievable; those providing an independent assessment of healing were blind to treatment allocation. Treating clinicians were expected to (not required to) use the randomised sampling strategy throughout.

### 2.3 Interventions

*In accordance with current UK guidelines, s*taff were required to clean and debride the wound prior to sampling. Participants were to have the index DFU (largest at baseline) and all other DFUs sampled by the randomised method.

All samples were delivered to local microbiology laboratories for routine culture and sensitivity (C&S) as soon as possible. A second sample was sent to a central laboratory for molecular analyses (reported separately). Swab sampling for C&S used a cotton‐tipped swab rubbed over the surface, with enough pressure to capture fluids from within the ulcer.^8^ Tissue sampling used a dermal curette or scalpel blade to collect tissue from the base of the wound in a standardised way. Training and guidance were provided during site induction, which included videos and written instructional materials. The videos were also hosted on our dedicated website, accessible only to sites, to allow for refresher sessions or review as needed.

### 2.4 Assessments and Outcome Measures

Baseline assessments were conducted in clinic, prior to randomisation and initiation of the randomised sampling strategy (swab or tissue). These included clinical history: diabetes type, duration of diabetes, number of ulcers on infected foot/both feet, whether ischaemic/neuropathic/neuro‐ischaemic, aetiology, and recent HbA1C result (within 3 months). Also recorded were current therapies: current and proposed antibiotic treatment (agent and dates), type of diabetes management (e.g. insulin, oral hypoglycaemic agents, other non‐insulin injectables', diet alone), and primary wound dressing. Finally, we recorded index ulcer characteristics: first or recurrent ulcer, duration of index ulcer, site of index ulcer, depth, neuropathy by 10 g monofilament, presence of ischaemia (by palpating pulses and/or presence of multiphase doppler signal by hand held doppler) and severity of infection by IDSA criteria, and index wound area by acetate tracing and measurement.

Participants were assessed in person at weeks 4, 12, and 26, with subsequent remote assessments at weeks 39, 52, and 104 (postal quality of life, or medical record review). Participants recruited in the first year of recruitment were followed up to 104 weeks; others for 52 weeks. Assessments in person were for wound tracing and photography (at week 4 only), quality of life (EQ‐5D‐3L, DFS‐SF and healthcare resource use questionnaires, up to week 26), and clinical checks for index DFU healing. At all visits, records were reviewed for reports of amputation, osteomyelitis, hospitalisations, additional sampling, and antibiotic prescriptions. Throughout follow‐up, participants' ulcers were reviewed, with any report of healing to be confirmed within a target of 3 days of initial identification by a blinded assessor.

The primary outcome measure was the time to healing of the index DFU, with blinded confirmation within 14 days of the initial report (or central photo adjudication). The protocol required blinded confirmation within 3 days of the initial report, with a longer timeframe for analysis agreed to allow for unexpected delays. Healing was defined as complete epithelialisation of the ulcer maintained for 14 days. Time to healing was assessed in the presence of the competing risks of death (all causes) and amputation. A sensitivity analysis allowed for any report of healing to be counted regardless of confirmation, in the presence of competing risks. Secondary outcome measures included: proportion compliant with randomised sampling at baseline/follow‐up; antibiotic utilisation; reduction in DFU area at 4 weeks; duration of hospitalisation for DFU reasons; and time to death.

### 2.5 Statistical Methods

The planned sample size of 730 participants provided 90% power for detecting a target effect size of 12.5% difference in the proportion of participants with healing of their index DFU at 52 weeks post‐randomisation, based on centred rate of 45%.^11^ This assumes a 2‐sided 5% significance level and 10% attrition. In all analyses, participants were analysed in their randomised groups, with all participants included in primary outcome measure analyses, and remaining analyses and summaries using those with complete data only with no imputation of missing outcome data. The primary outcome measure was analysed using Zhou et al's extension to the Fine and Gray sub‐distribution hazards model to competing risks data, allowing for within‐centre clustering and adjusting for the minimisation variables.^15^, ^16^ Time‐varying effects and/or non‐linear terms were explored, with the final primary model including square root transformations of DFU area and duration (but no time‐varying effects) and the sensitivity analysis model including a square root term for DFU duration and time‐varying effects for square root transformed DFU duration and area. Percentage reduction in DFU area at 4 weeks post‐randomisation included participants with complete data in 4‐week assessments within 21–35 days of randomisation. All secondary outcome measures were summarised descriptively.

### 2.6 Changes to study objectives, outcome measures and analyses

The trial's objectives and analyses underwent major revision following early closure of the study for futility of recruitment. Objectives were re‐stated in terms of “estimating” and “exploring” effects of interventions on outcomes. For the primary outcome measure ‘Time to ulcer healing’, the requirement for blinded in person confirmation of healing was relaxed to permit a central photography review to substitute for blinded adjudication. The originally planned analysis removed the requirement to account for the time‐varying effect of uptake of surgical revascularisation. Following an unexpected baseline imbalance, exploratory sample‐hoc analyses adjusting for the effect of age were conducted.

## 3 RESULTS

**FIGURE 1:**
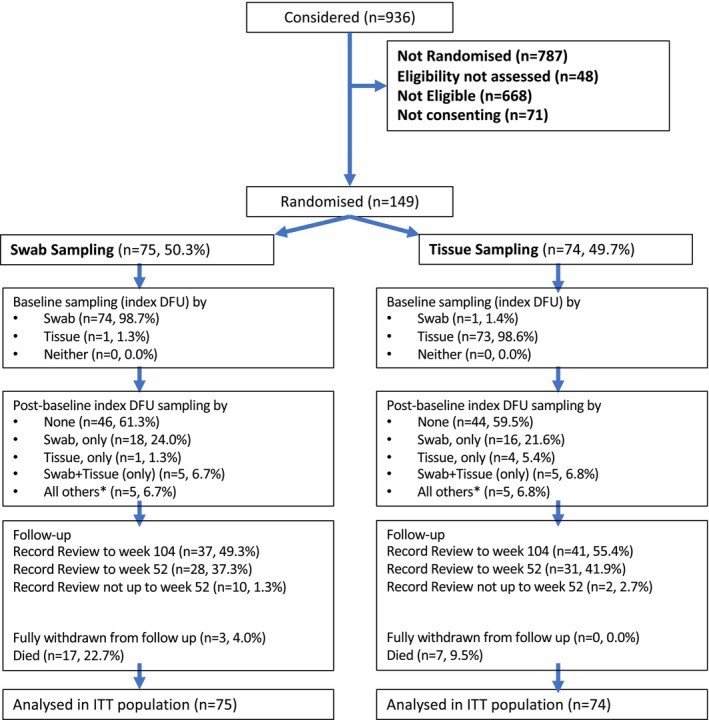
CONSORT flow diagram. *All other combinations of sampling methods, with or without swab/tissue sampling.

The CONSORT flow diagram (Figure 1) summarises the recruitment and participant flow. Between 1st May 2019 and 3 May 2022, 936 patients across 21 sites were considered for inclusion. Of these, 668 (71.4%) were ineligible, mainly due to suspected osteomyelitis (271, 40.6%), severe foot infection (92, 13.8%) or being unsuitable for tissue sampling (64, 9.6%). Overall, 149 (67.7% of those eligible) participants were randomised between 7th May 2019 and 28th April 2022. No one was recruited between 14th March 2020 and 30th July 2020 due to the COVID‐19 pandemic. The trial closed to recruitment on 19th May 2022 after the recruitment of 149 participants.

The groups were similar in most respects; however, participants in the swab group were on average 6 years older than the tissue group (mean (range) was 65.7 (32–93) and 59.7 (31–86) for swab vs. tissue groups, respectively) and had a slightly higher proportion of males (86.7% vs. 78.4% respectively), see Table 1. The median (inter quartile range (IQR)) index ulcer areas were 2.2 cm^2^ (0.7–4.7 cm^2^) and 1.1 cm^2^ (0.5–3.1 cm^2^) in swab and tissue groups, respectively. Almost all participants were of white ethnicity (96.6%), had Type II diabetes (87.9%), and a median (IQR) duration of diabetes of 16 (10–22) years. For 79.2% of participants, the index ulcer was their first‐ever ulcer; only 27.5% had more than one ulcer at baseline, and the median (IQR) duration of the index ulcer was 1 (0.5–4) month. Most index ulcers were neuropathic (86.6%) and had moderate (IDSA) infection (66.4%).^6^ Seventy‐two (48.3%) were receiving antibiotics and/or antimicrobial/antiseptic dressings at baseline.

**TABLE 1:** Baseline characteristics.

	Swab sampling (*n* = 75)	Tissue sampling (*n* = 74)	Total (*n* = 149)
Age, years	65.7 (11.39)	59.7 (12.98)	62.7 (12.54)
[Minimum, Maximum]	[32, 93]	[31, 86]	[31, 93]
Sex, male	65 (86.7%)	58 (78.4%)	123 (82.6%)
Ethnicity, white	72 (96.0%)	72 (97.3%)	144 (96.6%)
Type II Diabetes	68 (90.7%)	63 (85.1%)	131 (87.9%)
Duration of diabetes (years) median [IQR]	15.0 [10.0–24.0]	17.0 [11.0–21.0]	16.0 [10.0–22.0]
HbA1c, mmol/mol	70.1 (22.97)	73.3 (24.09)	71.7 (23.49)
Current treatment for diabetes			
Oral hypoglycaemic agent	52 (69.3%)	45 (60.8%)	97 (65.1%)
Insulin	37 (49.3%)	47 (63.5%)	84 (56.4%)
Other non‐insulin injectables	8 (10.7%)	3 (4.1%)	11 (7.4%)
Diet alone	9 (12.0%)	2 (2.7%)	11 (7.4%)
DFU on both feet	9 (12.0%)	8 (10.8%)	17 (11.4%)
More than one ulcer at baseline	21 (28.0%)	20 (27.0%)	41 (27.5%)
Index DFU initial (non‐recurrent)	59 (78.7%)	59 (79.7%)	118 (79.2%)
Index DFU aetiology			
Neuro‐ischaemic	10 (13.3%)	7 (9.5%)	17 (11.4%)
Ischaemic	—	2 (2.7%)	2 (1.3%)
Neuropathic	64 (85.3%)	65 (87.8%)	129 (86.6%)
No neuropathy or ischaemia (unusual presentation)	1 (1.3%)	—	1 (0.7%)
Index DFU located on forefoot (± digits)	63 (84.0%)	61 (82.4%)	124 (83.2%)
Duration of index DFU (months) median [IQR]	1.0 [0.5–3.0]	2.0 [0.5–4.0]	1.0 [0.5–4.0]
Index ulcer grade			
Grade 1—Superficial full‐thickness	46 (61.3%)	52 (70.3%)	98 (65.8%)
Grade 2—Deep ulcer, penetrating to below dermis	26 (34.7%)	15 (20.3%)	41 (27.5%)
Grade 3—Affecting all layers, including bone and/or joint	3 (4.0%)	7 (9.5%)	10 (6.7%)
Index DFU area (cm^2^) median [IQR]	2.2 [0.7–4.7]	1.1 [0.5–3.1]	1.3 [0.6–3.8]
Infection classification			
Mild	53 (70.7%)	46 (62.2%)	99 (66.4%)
Moderate	22 (29.3%)	27 (36.5%)	49 (32.9%)
Grade 4 severe	—	1 (1.4%)	1 (0.7%)
Prior treatments			
Both antimicrobial/antiseptic dressings and antibiotics (any indication)	9 (12.0%)	9 (12.2%)	18 (12.1%)
Antibiotics (any indication) only	12 (16.0%)	10 (13.5%)	22 (14.8%)
Antimicrobial/antiseptic dressings only	17 (22.7%)	15 (20.3%)	32 (21.5%)
Neither	37 (49.3%)	40 (54.1%)	77 (51.7%)

*Note*: Values are either mean (standard deviation) or *n* (percentage), unless otherwise stated.

**FIGURE 2:**
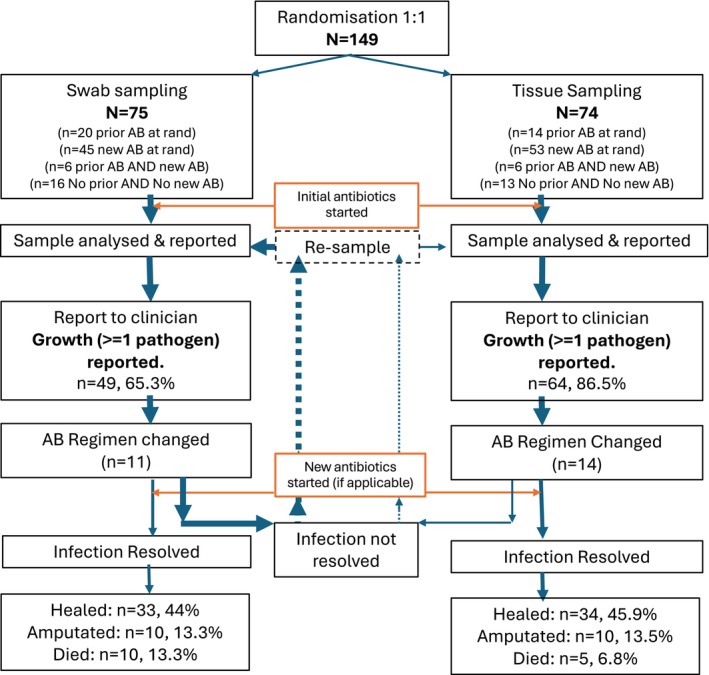
Summary of CODIFI2 test‐treat model and outcome. Proportions of reports with 1 or more pathogens exclude 2 from each arm with no clinical review results.

**FIGURE 3:**
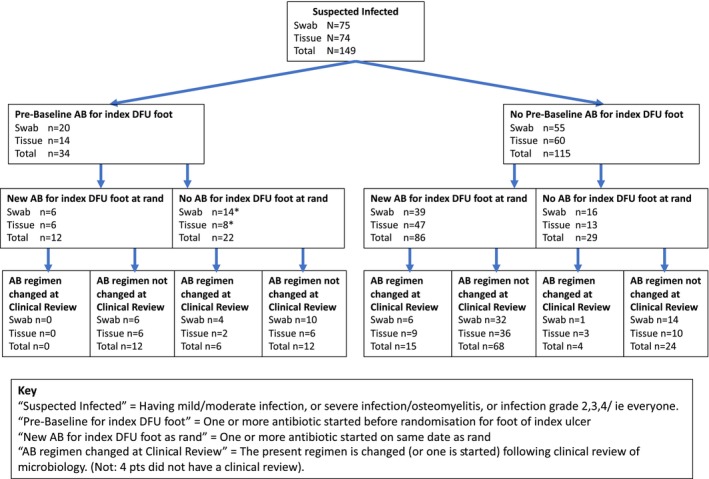
Summary of the timing of the first AB prescription and subsequent changes.

Baseline microbiology showed 49/75 (65.3%) swab and 64/74 (86.5%) tissue reports described at least one bacterial organism (Figure 2) At clinical review, 11/73 swab and 14/72 tissue participants (15.1% vs. 19.4%) had their antibiotic regimen changed (Figure 3). Twenty‐four participants (16.1%) (14 swab, 10 tissue) had no antibiotics prescribed pre‐randomisation, at randomisation, or at clinical review. Participants randomised to tissue sampling had slightly more courses of antibiotics prescribed during follow‐up (median (IQR) 2 (1–4) and 3 (1–6) for swab vs. tissue, respectively). The index DFU was typically sampled a median (IQR) of 1 (1–2) time post‐randomisation in both groups. One participant in each group (1.3% swab vs. 1.4% tissue) had their index DFU sampled using the non‐randomised method at baseline, and the incidence of non‐randomised sampling was higher for tissue compared to swabbing: by 52 weeks, 16.4% of participants in the swab arm crossed over to tissue and/or bone sampling, compared to 29.9% in the tissue arm, who crossed over to swab or bone sampling.

### 3.1 Primary Outcome Measure

Thirty‐three swab and thirty‐four tissue participants (44.0% and 45.9% respectively) had confirmed ulcer healing as their first event for the primary outcome measure at the end of their follow‐up. Table 2 summarises the cumulative incidences of confirmed healing and competing first events at pre‐defined study timepoints by arm. The cumulative incidences of healing at 52 weeks—allowing for censoring and competing events—were 45.3% (95% CI 33.5%–56.4%) and 44.6% (33.0%–55.6%) for swab and tissue groups respectively. The hazard ratio (HR) for healing in those randomised to tissue vs. swab was 1.01 (95% CI 0.65–1.55). Adjusting for age made little difference to the estimated effect on healing (HR 0.98, 95%CI 0.60–1.60).

**TABLE 2:** Summary of first event (primary outcome analyses) by arm over time.

Event	Timepoint (weeks)	Cumulative incidence (%, swab)	95% CI for CIF (swab)	Cumulative incidence (%, tissue)	95% CI for CIF (tissue)	Cumulative incidence (%, all)	95% CI for CIF (all)
Healed (primary)	12	16.2	8.9%–25.5%	20.3	12.0%–30.1%	18.2	12.5%–24.9%
	26	32.7	22.3%–43.6%	36.5	25.6%–47.4%	34.6	27.0%–42.3%
	39	42.4	30.8%–53.4%	43.2	31.7%–54.2%	42.8	34.7%–50.6%
	52	45.3	33.5%–56.4%	44.6	33.0%–55.6%	44.9	36.7%–52.8%
	104	45.3	33.5%–56.4%	44.6	33.0%–55.6%	44.9	36.7%–52.8%
Amputation involving index DFU	12	6.8	2.5%–14.0%	6.8	2.5%–14.0%	6.8	3.4%–11.6%
	26	12.2	6.0%–20.9%	10.8	5.0%–19.1%	11.5	7.0%–17.3%
	39	12.2	6.0%–20.9%	12.2	5.9%–20.8%	12.2	7.5%–18.1%
	52	12.2	6.0%–20.9%	13.5	6.9%–22.4%	12.9	8.1%–18.9%
	104	13.9	7.1%–23.1%	13.5	6.9%–22.4%	13.8	8.7%–20.0%
Died, any causes	12	1.4	0.1%–6.5%	0.0	—	0.7	0.1%–3.4%
	26	4.1	1.1%–10.5%	0.0	—	2.0	0.6%–5.4%
	39	8.2	3.3%–16.0%	1.4	0.1%–6.6%	4.8	2.1%–9.1%
	52	8.2	3.3%–16.0%	4.1	1.1%–10.5%	6.1	3.0%–10.8%
	104	15.4	7.7%–25.4%	7.8	2.8%–16.2%	11.6	6.7%–17.9%

*Note*: For the primary analysis, healing of index DFU (“healed” required blinded confirmation of healing within 14 days of initial report or adjudication of healing from central photography review).

Abbreviations: CIF, cumulative incidence function; DFU, diabetic foot ulcer.

**FIGURE 4:**
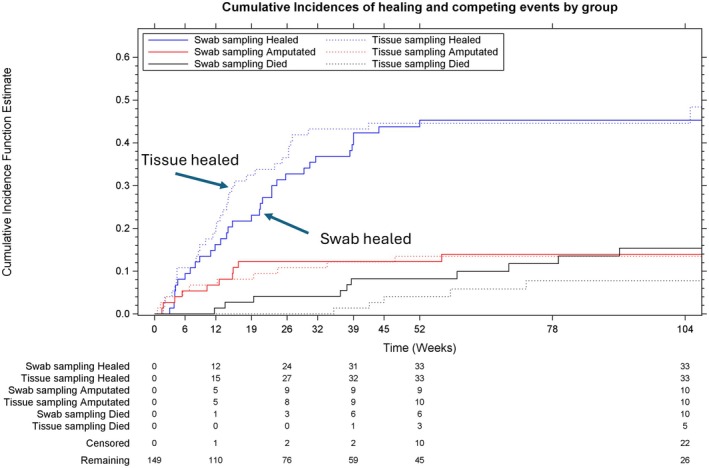
Time to confirmed ulcer healing with competing risks of amputation and death.

**FIGURE 5:**
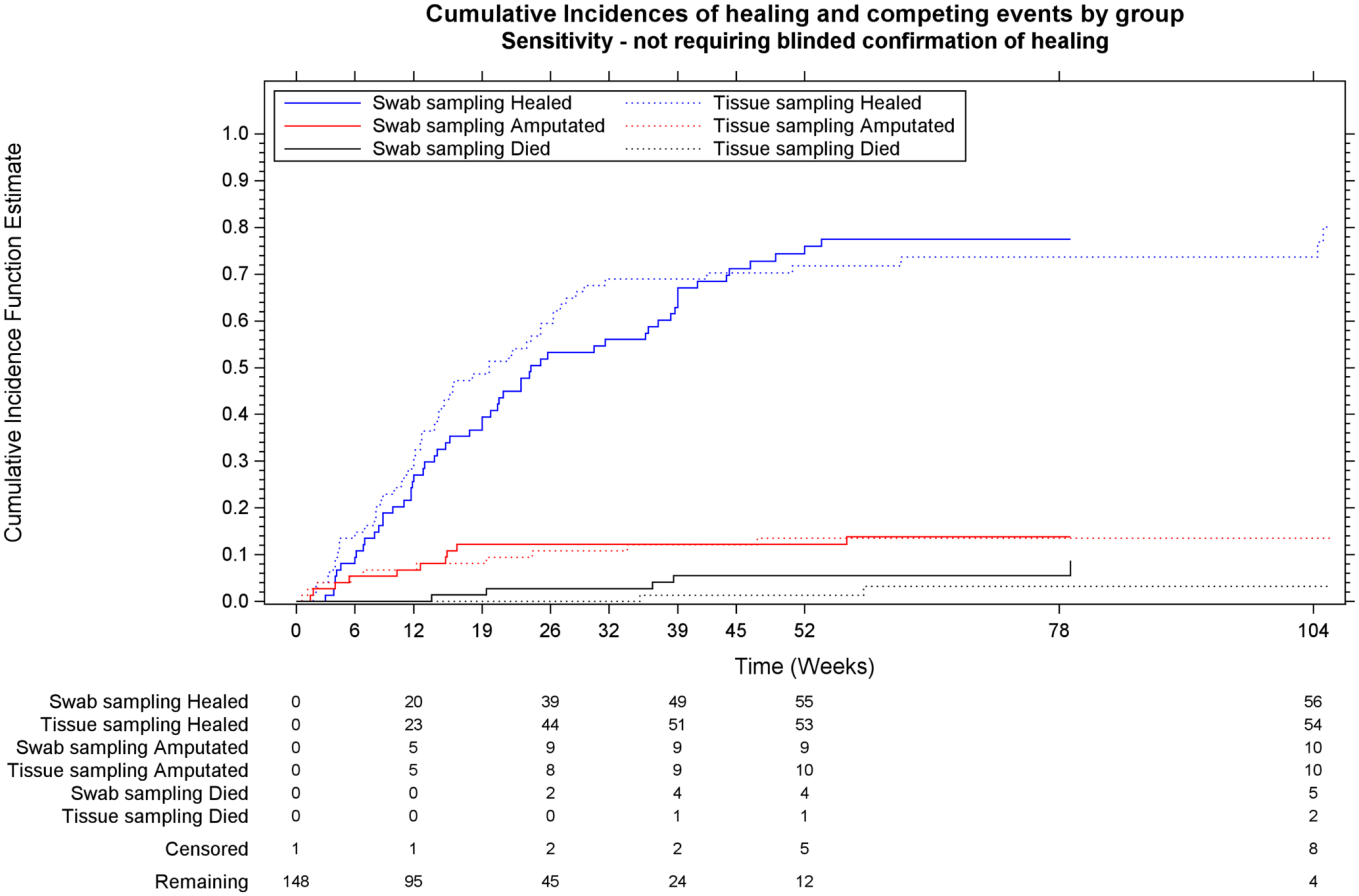
Time to report of index ulcer healing—not requiring confirmation of healing—with competing risks of amputation and death.

Table 3 reports cumulative incidences of any report of healing and competing risk events over time (sensitivity analysis). The cumulative incidences of healing at 52 weeks—allowing for censoring and competing events—were 75.9% (95% CI 63.8%–84.5%) and 71.8% (59.7%–80.8%) for swab and tissue, respectively. The effect of randomisation to tissue vs. swab sampling on time to healing, in the final sensitivity analysis model, was (HR = 1.12, 95% CI 0.81–1.55). In the post‐hoc analysis, the effect was slightly reduced after adjusting for age (HR = 1.06, 95% CI 0.75–1.49). Figures 4 and 5 present the time to first event for the primary outcome measure analysis under the primary outcome measure definition (Figure 4, confirmed healing) and the sensitivity analysis (Figure 5, reported healing).

**TABLE 3:** Summary of first event (primary outcome measure sensitivity analysis) by arm over time.

Event	Timepoint (weeks)	Cumulative incidence (%, swab)	95% CI for CIF (swab)	Cumulative incidence (%, tissue)	95% CI for CIF (tissue)	Cumulative incidence (%, all)	95% CI for CIF (all)
Healed (primary)	12	27.0	17.4%–37.5%	31.1	20.9%–41.8%	29.1	21.9%–36.5%
	26	53.2	41.1%–64.0%	59.5	47.2%–69.8%	56.4	47.9%–64.0%
	39	67.1	54.8%–76.7%	68.9	56.8%–78.3%	68.0	59.7%–74.9%
	52	75.9	63.8%–84.5%	71.8	59.7%–80.8%	73.7	65.7%–80.2%
	104	77.5	65.5%–85.8%	73.7	61.4%–82.6%	75.6	67.5%–82.0%
Amputation involving index DFU	12	6.8	2.5%–14.0%	6.8	2.5%–14.0%	6.8	3.4%–11.6%
	26	12.2	6.0%–20.9%	10.8	5.0%–19.2%	11.5	7.0%–17.3%
	39	12.2	6.0%–20.9%	12.2	5.9%–20.8%	12.2	7.5%–18.1%
	52	12.2	6.0%–20.9%	13.5	6.8%–22.5%	12.9	8.1%–18.9%
	104	13.8	6.9%–23.2%	13.5	6.8%–22.5%	13.8	8.7%–20.1%
Died, any causes	12	0.0	—	0.0	—	0.0	—
	26	2.8	0.5%–8.7%	0.0	—	1.4	0.3%–4.5%
	39	5.5	1.7%–12.6%	1.4	0.1%–6.8%	3.4	1.3%–7.4%
	52	5.5	1.7%–12.6%	1.4	0.1%–6.8%	3.4	1.3%–7.4%
	104	8.7	2.2%–20.8%	3.3	0.5%–10.7%	5.6	2.3%–10.9%

*Note*: In the sensitivity analysis, healing required only a report of healing. Blinded confirmation or photo adjudication was not required.

### 3.2 Secondary Outcome Measures

Table 3 presents the summary results for the secondary outcome measures.

A week‐4 assessment was available in 109 participants, and the median (IQR) percentage reduction in area was 55.4% (17.9%–89.3%) for the swab group vs. 61.6% (11.8%–90.2%) for the tissue group.

Over the follow‐up, 17/75 (22.7%) swab and 7/74 (9.5%) tissue participants died. Table 4 summarises the proportions of participants dying at each timepoint, when healing of the index DFU and amputations are not considered. The hazard ratio for time to death (tissue vs. swab, adjusted for age) was 0.44 (95% CI 0.18–1.08).

**TABLE 4:** Summary of secondary outcome measure results.

Outcome measure	Swab	Tissue
Reduction in ulcer area at 4 weeks	(*n* = 57)	(*n* = 52)
Median	55.4%	61.6%
(Interquartile range)	(17.9%–89.3%)	(11.8%–90.2%)
50% reduction or more	34 (59.6%)	29 (55.8%)
100% reduction	6 (10.5%)	8 (15.4%)
Death	(*n* = 75)	(*n* = 74)
Died within 12 weeks	1 (1.3%)	0 (0.0%)
Died within 26 weeks	3 (4.0%)	0 (0.0%)
Died within 39 weeks	6 (8.0%)	1 (1.4%)
Died within 52 weeks	8 (10.7%)	3 (4.1%)
Died within 104 weeks	17 (22.7%)	7 (9.5%)
	(*n* = 21)	(*n* = 25)
Hospitalisation for DFU reasons, median % age time admitted	2.8%	3.0%
(Interquartile range)	(2.1%–6.5%)	(1.6%–4.7%)
Median days admitted	17	16
(Interquartile range)	(12–39)	(10–32)
Proportion of time prescribed antibiotics for index ulcer foot, median	6.1%	8.8%
(Interquartile range)	(2.1%–16.7%)	(4.0%–18.5%)
Non‐serious adverse events		
Participants %, (*n* events)	47 (62.7%), [131 events]	59 (79.7%), [195 events]
Sampling Method related (events)		
pain	3	1
Bleeding	—	15
New/recurrent DFU on index foot	52	68
New infection on index foot	46	67
Osteomyelitis on either lower limb	22	23
AE related to antibiotics	8	21
Related and expected serious adverse events		
Participants %, (*n* events)	21 (28.0%), [43 events]	25 (33.8%), [54 events]
Hospitalised related to index DFU	5	5
Hospitalised related to non‐index DFU	19	26
Osteomyelitis	9	11
Cellulitis	4	10
Worsening Infection	3	3
Septicaemia and all other	3	2
Amputations, (events)	19	23
Unrelated and expected serious adverse events		
Participants (%), [*n* events]	34 (45.3%), [59 events]	20 (27.0%), [38 events]
Death	17	7
Hospitalisation for other causes	42	31

The median (IQR) total duration of admissions, in days, was 17 (12–39) and 16 (10–32) and the median (IQR) proportion of time hospitalised while on study was 2.8% (2.1%–6.5%) 3.0% (1.6%–4.7%) for swab and tissue sampling groups respectively, with 21 swab and 25 tissue participants admitted to hospital at least once for a DFU reason (Table 4).

A total of 131 related and expected non‐serious adverse events were reported in 47 swab participants and 195 events in 59 tissue sampling participants. The difference was largely accounted for by the 15 reports of minor bleeding due to sampling in tissue participants, an additional 16 new or recurrent DFU, 21 additional new episodes of infection, and 13 additional adverse events related to antibiotics. Forty‐three related and expected serious adverse events (amputation, index DFU related hospitalisation and hospitalisation due to a non‐index DFU) were reported in 21 swab participants and 54 events in 25 tissue participants. Overall, 14 swab group participants (18.7%) underwent 19 amputations (major and minor) and 18 tissue participants (24.3%) underwent 23 amputations (major and minor) across either limb. Table S1 includes further detail on the extent, timing, and location of the limb affected by amputation. Moreover, 59 unrelated and expected serious adverse events (deaths and acute hospitalisations) for other non‐DFU causes were reported in 34 swab group participants and 38 events in 20 tissue group participants. There were no related and unexpected serious adverse events.

## 4 DISCUSSION

Although tissue sample reports identified at least one pathogen more often than swabbing (86.5%, 65.3% respectively), this did not translate into a large difference in clinician behaviour: 19.4% and 15.1% in tissue and swab, respectively, had a change in antibiotics. There were small differences in 4‐week area changes and no clear difference in the primary outcome of time to ulcer healing or proportions of ulcers healed at 52 weeks, although the trial was much smaller than intended and hence is under‐powered.

The difference in the cumulative incidence of healing observed in the study was less than 1% (45.3% and 44.6% for swab and tissue respectively). The corresponding hazard ratio for time to healing tissue vs. swab sampling was 1.01 (95% CI 0.65–1.55), and although there is low precision in this estimate, we conclude there is no evidence of a difference in time to healing between sampling methods.

Strengths of this trial include the use of clinically important and objective outcomes of healing and amputation, rather than ‘resolution of infection’ or agreement between sampling approaches. It was allocation blind and pragmatic, with a primary objective to determine the clinical effectiveness of a *policy* of either tissue or swab sampling in terms of time to healing in patients with a suspected DFU infection throughout their ulcer treatment, with sufficient duration of follow‐up (at least 52 weeks) to capture clinically important events of healing, amputation, and death. CODIFI2 also collected confirmed healing date (using a blinded assessor) with a sensitivity analysis using unconfirmed healing supplemented with remote photographic confirmation, to mitigate effects of observer bias.

The trial design, which allowed for repeated sampling and review, is a *test‐treatment trial* requiring the full analysis set of all participants randomised.^17^ This contrasts with the ‘traditional’ diagnostic accuracy study design, in which a single baseline test is used to change treatment and outcome; such designs are based on different sample size assumptions.^18^ Microbiological sampling is not a ‘diagnostic test for infection’ as the diagnosis of infection in DFU is primarily clinical.^6^ Microbiological information facilitates accurate antibiotic treatment by allowing for adjustments based on laboratory results and clinical review. We have not been able to identify any previous trials of sampling approaches with clinical and cost‐effectiveness outcomes.

Overall, the study was too small for significance testing for a number of clinical outcomes, but the raw data reported more participants with pathogens reported by tissue collection than swab, a higher number of pathogens identified with tissue, and more antibiotics prescribed for the tissue group.

The small difference in antibiotic prescriptions may indicate that the higher yield from tissue samples drives more antibiotic prescribing, but this did not translate into evidence of increased DFU healing. The prescription change rate observed here is smaller than the theoretical change rate in a previous study^11^ and this demonstrates the importance of research into actual, rather than reported, behaviour.

Whilst participants were included based on suspected clinical infection of their DFU according to IDSA criteria,^6^ 16.1% had no antibiotics prescribed before randomisation or at first review. This suggests either that clinicians were more uncertain about the clinical diagnosis of infection than anticipated, or that the criteria used for the diagnosis may be less specific than previously thought. Our finding that not all enrolled participants were being clinically managed with antibiotics,^4^ may have reduced the power of the study. The higher cross‐over rate from tissue to swab than vice versa may indicate that swab samples are used as a default when time or resources are short, or are perceived as easier.

Despite more organisms reported and higher use of antibiotics for tissue, there was no large effect on the number of or time to healing events, on amputations, or death.

There were more adverse events with tissue sampling, mainly minor bleeding.

The best practice guidelines that recommend tissue sampling as it provides a higher yield than swab sampling, which is supported by our findings too, but the actual impact upon clinical outcome has not previously been tested in a pragmatic trial. Those who advocate tissue sampling may argue that it provides clinical information on the wound biome not only for the tailoring of treatment for the current episode of care, but also for future DFU episodes of infection, as well as for understanding the population of DFU infection locally. It could be argued, however, that our trial of a policy of swab versus tissue, allowing for repeated use of the allocated method, sought to capture any benefit from this wider knowledge of infection and sensitivity patterns. It may be that a much larger sample size would be required to identify any such benefits.

Furthermore, these findings may mean that the role of microbiological sampling of mild or moderately infected DFU is less impactful than previously considered. This could be because antibiotics are started at initial identification and hence the receipt of microbiology results 3–5 days later, describing a wound microbiome now altered by the prescription of antibiotics, rendering the utility of the initial sample reduced, has a very modest scope for improving treatment of infection. This may be related to the implementation of well‐developed protocols for the care of infected DFU in these populations in the UK settings recruiting to CODIFI2, with clinical outcomes on a par with those in the UK National Diabetes Foot Audit.^3^ As such, there was less opportunity for the microbiology results to impact most patients' care as prescribing was already based upon local expert knowledge of likely pathogens and sensitivities, and hence there were few opportunities for improvement in that particular aspect of care from different sampling. There may be a high therapeutic inertia associated with reviewing and adjusting antimicrobials in these patients, particularly if there is any clinical improvement despite a microbiology report identifying potential scope for antimicrobial tailoring. Whether this narrow opportunity for benefit capture applies in other DFU care settings, and especially other wound types, is not known.

Unanswered questions from this study include the reasons for around 1 in 8 people considered to have a clinically infected DFU not being prescribed antibiotics. Given the small difference in outcomes for the two forms of sampling, a follow‐on question is whether there is a benefit associated with microbiology sampling for individual patient care in this population, given that antibiotic prescriptions do not depend upon sampling results. There may be a clinical perception that additional information from wound tissue sampling, resulting in more reported organisms, is ‘better’, despite it being associated with more adverse events such as minor bleeding and higher costs, with little impact upon both treatment decisions and clinical outcomes. The impact of these findings on our responsibilities for antimicrobial stewardship is also worthy of further investigation. One implication of these results might be a move towards more swab sampling, and whether this leads to any change in the use of broad‐spectrum antimicrobials, as opposed to targeted therapeutic agents, should be monitored.

## FUNDING INFORMATION

This study was funded by the UK National Institute for Health Research Health Technology Assessment Programme. It was a commissioned call for research (reference 16/163/04), the funders specified the study population and sample, the intervention, and the primary outcomes. The sponsor was the University of Leeds. The sponsor and funder were not involved in the design of the study; the collection, analysis, and interpretation of data; writing the report; and did not impose any restrictions regarding the publication of the report.

## CONFLICT OF INTEREST STATEMENT

EAN, TJS, FG, RG, HK, DR, AO, MB have no declarations regarding industry connections or other personal connections that may be perceived to have influenced this work.

## Supporting information


Table S1.

## Data Availability

Anonymised data will be available on request from the authors for non‐commercial purposes. Further data supporting the results of this article can be found in the NIHR synopsis.
