# Supplementary material for: CODIFI2: Randomised controlled trial to compare clinical and cost‐effectiveness of swabs versus tissue sampling to inform management of infected diabetic foot ulcers
Source: Diabet Med. 2025 Mar 6;42(5):e70009. doi: 10.1111/dme.70009 (PMC12006554; doi:10.1111/dme.70009)
Supplement: Supplementary file 1 — Table S1. [file DME-42-e70009-s001.docx]

**SUPPLEMENTARY TABLE X (1)**

**Further summary of amputation procedures**

|  | **Swab sampling** | **Tissue sampling** | **Total** |
| --- | --- | --- | --- |
| **All amputations** | **19 (14pts)** | **23 (18pts)** | **42 (32pts)** |
| Amputations occurring before confirmed healing (incl amputations where healing not confirmed) | 16 (13pts) | 19 (14pts) | 35 (27pts) |
| Amputations occurring after confirmed healing | 3 (3pts) | 4 (4pts) | 7 (7pts) |
|  |  |  |  |
| **Amputations on the limb of the index ulcer** | **16 (14pts)** | **21 (17pts)** | **37 (31pts)** |
| ***Minor/major*** |  |  |  |
| Amputations on the limb of the index ulcer (Major) | 5 (5pts) | 9 (8pts) | 14 (13pts) |
| Amputations on the limb of the index ulcer (Minor) | 11 (10pts) | 12 (9pts) | 23 (19pts) |
| ***Whether involving index DFU*** |  |  |  |
| Amputations on the limb of the index ulcer (Involving index DFU) | 11 (11pts) | 10 (10pts) | 21 (21pts) |
| Amputations on the limb of the index ulcer (Not involving index DFU) | 5 (5pts) | 11 (9pts) | 16 (14pts) |
|  |  |  |  |
| **Amputations on the Contralateral limb** | **3 (3pts)** | **2 (2pts)** | **5 (5pts)** |
| Amputations on the contralateral limb (Major) | - | - | - |
| Amputations on the contralateral limb (Minor) | 3 (3pts) | 2 (2pts) | 5 (5pts) |

Footnote: In each cell, the value is the number of events and (in brackets) the number of participants with one or more such event.

**SUPPLEMENTARY TABLE X (2)**

**Time to event model fit for time to confirmed DFU healing, in presence of competing risks of amputation and death.**

|  | **Hazard Ratio** | **95% Confidence interval for Hazard Ratio** |
| --- | --- | --- |
| **Randomised to TISSUE sampling vs swab sampling** | 1.01 | (0.65 to 1.55) |
| ISCHAEMIC Aetiology, vs Neuropathic | 0.78 | (0.37 to 1.63) |
| NEURO-ISCHAEMIC Aetiology, vs Neuropathic | 0.30 | (0.08 to 1.13) |
| NORMAL Aetiology, vs Neuropathic | 4.90 | (1.97 to 12.16) |
| Per extra unit of sqrt(cm^2^) of index DFU area | 0.63 | (0.45 to 0.87) |
| Per extra unit of sqrt(duration in months) | 0.67 | (0.44 to 1.02) |
| Per extra ulcer observed at baseline | 1.10 | (0.66 to 1.85) |
| MID/HINDFOOT location vs Forefoot | 1.18 | (0.60 to 2.29) |

**Note, hazard ratios are relative to that for a participant randomised to Swab sampling with a neuropathic DFU on the forefoot, no additional ulcers beyond the index ulcer, and the index ulcer having the same area and duration as the mean.**
